# Incidence of community acquired lower respiratory tract disease in Bristol, UK during the COVID-19 pandemic: A prospective cohort study

**DOI:** 10.1016/j.lanepe.2022.100473

**Published:** 2022-08-08

**Authors:** Catherine Hyams, Robert Challen, Elizabeth Begier, Jo Southern, Jade King, Anna Morley, Zsuzsa Szasz-Benczur, Maria Garcia Gonzalez, Jane Kinney, James Campling, Sharon Gray, Jennifer Oliver, Robin Hubler, Srinivas Valluri, Andrew Vyse, John M. McLaughlin, Gillian Ellsbury, Nick A. Maskell, Bradford D. Gessner, Leon Danon, Adam Finn, Avon CAP Research Group, Amelia Langdon, Anabella Turner, Anya Mattocks, Bethany Osborne, Charli Grimes, Claire Mitchell, David Adegbite, Emma Bridgeman, Emma Scott, Fiona Perkins, Francesca Bayley, Gabriella Ruffino, Gabriella Valentine, Grace Tilzey, Johanna Kellett Wright, Julia Brzezinska, Julie Cloake, Katarina Milutinovic, Kate Helliker, Katie Maughan, Kazminder Fox, Konstantina Minou, Lana Ward, Leah Fleming, Leigh Morrison, Lily Smart, Louise Wright, Lucy Grimwood, Maddalena Bellavia, Madeleine Clout, Marianne Vasquez, Milo Jeenes-Flanagan, Natalie Chang, Niall Grace, Nicola Manning, Oliver Griffiths, Pip Croxford, Peter Sequenza, Rajeka Lazarus, Rhian Walters, Robin Marlow, Robyn Heath, Rupert Antico, Sandi Nammuni Arachchge, Seevakumar Suppiah, Taslima Mona, Tawassal Riaz, Vicki Mackay, Zandile Maseko, Zoe Taylor, Zsolt Friedrich

**Affiliations:** a Bristol Vaccine Centre, Population Health Sciences, University of Bristol, UK; b Academic Respiratory Unit, University of Bristol, UK; c Engineering Mathematics, University of Bristol, UK; d Global Medical Development Scientific and Clinical Affairs, Pfizer Vaccines, Ireland; e Vaccines Medical Affairs, Pfizer Ltd, Tadworth KT20 7NS, UK; f Clinical Research and Imaging Centre, UHBW NHS Trust, Bristol, UK; g Vaccines Medical Development, Scientific and Clinical Affairs, Pfizer Inc, Collegeville, PA, USA

**Keywords:** Pneumonia, Lower respiratory tract infection, Cardiac failure, COVID-19, SARS-CoV-2, aLRTD, acute lower respiratory tract disease, COVID-19, Coronavirus disease 2019, CAP, community acquired pneumonia, COPD, chronic obstructive pulmonary disease, NP-LRTI, non-pneumonic lower respiratory tract infection, HF, heart failure, CRDE, chronic respiratory disease exacerbation

## Abstract

**Background:**

The emergence of COVID-19 and public health measures implemented to reduce SARS-CoV-2 infections have both affected acute lower respiratory tract disease (aLRTD) epidemiology and incidence trends. The severity of COVID-19 and non-SARS-CoV-2 aLRTD during this period have not been compared in detail.

**Methods:**

We conducted a prospective cohort study of adults age ≥18 years admitted to either of two acute care hospitals in Bristol, UK, from August 2020 to November 2021. Patients were included if they presented with signs or symptoms of aLRTD (e.g., cough, pleurisy), or a clinical or radiological aLRTD diagnosis.

**Findings:**

12,557 adult aLRTD hospitalisations occurred: 10,087 were associated with infection (pneumonia or non-pneumonic lower respiratory tract infection [NP-LRTI]), 2161 with no infective cause, with 306 providing a minimal surveillance dataset. Confirmed SARS-CoV-2 infection accounted for 32% (3178/10,087) of respiratory infections. Annual incidences of overall, COVID-19, and non- SARS-CoV-2 pneumonia were 714.1, 264.2, and 449.9, and NP-LRTI were 346.2, 43.8, and 302.4 per 100,000 adults, respectively. Weekly incidence trends in COVID-19 aLRTD showed large surges (median 6.5 [IQR 0.7–10.2] admissions per 100,000 adults per week), while other infective aLRTD events were more stable (median 14.3 [IQR 12.8–16.4] admissions per 100,000 adults per week) as were non-infective aLRTD events (median 4.4 [IQR 3.5–5.5] admissions per 100,000 adults per week).

**Interpretation:**

While COVID-19 disease was a large component of total aLRTD during this pandemic period, non- SARS-CoV-2 infection still caused the majority of respiratory infection hospitalisations. COVID-19 disease showed significant temporal fluctuations in frequency, which were less apparent in non-SARS-CoV-2 infection. Despite public health interventions to reduce respiratory infection, disease incidence remains high.

**Funding:**

AvonCAP is an investigator-led project funded under a collaborative agreement by Pfizer.

**Research in context**

**Evidence before this study**

Acute respiratory infection remains a leading worldwide cause of morbidity and mortality, with estimates of disease varying by population. The most recent prospective data from the UK were obtained before the emergence of SARS-CoV-2, estimating annual incidences of hospitalised community acquired pneumonia (CAP) for persons aged 65–74, 75–84, and ≥85 years of 1.6–3.1, 3.9–5.16, 5.1–15.2 per 1000, respectively. SARS-CoV-2 has changed the epidemiology of respiratory infection and, whilst there are extensive epidemiological data detailing hospitalisations of patients with COVID-19, there are few data describing either the total burden of acute lower respiratory tract disease (aLRTD) or respiratory infection due to other pathogens during the pandemic period. Some data suggest non-pharmaceutical measures implemented to reduce SARS-CoV-2 infection may also have reduced the burden of non-SARS-CoV-2 respiratory disease.

**Added value of this study**

We provide the first prospectively obtained description of total aLRTD in hospitalised adults covering the end of the first wave of the Wuhan strain, through subsequent waves of the Alpha and Delta variants and pre-dates the Omicron variant emergence. COVID-19 accounted for 32% of respiratory infections and COVID-19 disease was more severe than non-SARS-CoV-2 respiratory infection in persons aged >65 years. Further, while COVID-19 disease formed a large component of aLRTD during the pandemic, non-SARS-CoV-2 accounted for the majority of aLRTD hospitalisations. We therefore demonstrate that the burden of non-SARS-CoV-2 infection remained substantial, greater than SARS-CoV-2 in hospitalised adults, and was significantly higher than that previously estimated in the UK, even before the emergence of SARS-CoV-2.

**Implications of all the available evidence**

These results, highlight the importance of non-SARS-CoV-2 infection in contributing to the burden of aLRTD throughout the pandemic. In the context of an aging population with increasing comorbid disease, demonstrate that aLRTD remains an important public health concern and accounts for significant healthcare resources. Despite implementation of public health measures, both vaccination and non-pharmaceutical interventions, aLRTD incidence remained high and the incidence of non-COVID-19 disease may yet increase further. It is therefore essential that appropriate healthcare planning and resource allocation is undertaken to care for patients with aLRTD, in addition to implementation of public health measures to reduce respiratory disease burden and improve patient outcomes.

Alt-text: Unlabelled box

## Introduction

Respiratory infection is a leading cause of mortality and morbidity worldwide, with substantially higher disease in older and immunocompromised individuals. Data from the UK are relatively sparse. A population-based electronic database study conducted during 1997–2011 reported an average annual respiratory infection incidence of 123/1000 among UK adults aged ≥65 years, over half of whom were hospitalised, and a much lower incidence of pneumonia of 8.0/1000.^1^ More recently, a prospective observational cohort study conducted in Nottingham from 2013 to 2018 reported annual incidences of hospitalised community-acquired pneumonia (CAP) for persons aged 65–74, 75–84, and ≥85 years of 1.6–3.1, 3.9–5.16, 5.1–15.2 per 1000, respectively. Previous studies have used either radiological or microbiological diagnosis to define disease^2^^,^^3^ or retrospective collection of clinical-coding data^1^^,^^4^^,^^5^ to estimate incidence: both methods may have resulted in under-ascertainment of disease and its burden. Studies relying on typical signs and symptoms of respiratory infection may exclude elderly patients, who often present atypically and yet have the highest disease incidence.^6^ Furthermore, acute lower respiratory tract disease (aLRTD) also includes chronic respiratory disease exacerbation (CRDE) and heart failure (HF), which may co-exist with pneumonia and non-pneumonic lower respiratory tract infection (NP-LRTI). To provide appropriate healthcare resources and determine the effectiveness of public health interventions, including vaccinations against respiratory pathogens, it is essential that accurate data describing aLRTD disease phenotypes and incidences are generated and made available.

To address limitations of previous studies, the AvonCAP study prospectively and comprehensively captures data on all adults hospitalised with aLRTD within a defined geographical area. By ensuring all hospitalisations at study hospitals are screened for aLRTD using broad criteria, including patients presenting with atypical clinical features and those without a confirmed radiological or microbiological diagnosis, the study aims to capture aLRTD disease in its entirety. By conducting individual case assessment, hospital-acquired infection is excluded, and disease is accurately typed into subgroups including pneumonia, NP-LRTI, CRDE and HF.

As AvonCAP was preparing to start collecting data, the epidemiology of acute lower respiratory tract infection was changed in 2020–2021 by the COVID-19 pandemic, with large waves of admissions caused by the original strain of SARS-CoV-2 and successive variants, as well as changes in the incidence of many other respiratory infections resulting from the public health measures introduced to limit the pandemic. The emergence of COVID-19 resulted in unprecedented demand on healthcare resources and the UK, like many countries, implemented social distancing measures and a series of national lockdowns to reduce infections and hospitalisations.^7^ These measures probably also reduced transmission of other respiratory pathogens,^8^^,^^9^ and consequently may have affected respiratory infection hospitalisation rates. Recent studies suggest that both asthma and chronic obstructive pulmonary disease (COPD) related hospitalisations decreased following the emergence of COVID-19.^10^, ^11^, ^12^ Other factors may affect aLRTD admission rates among adults including clinician practice (including admission thresholds), and patient treatment preferences, which may have changed during the pandemic. The impact of these factors, in addition to the emergence of COVID-19, on total respiratory disease burden is unclear.

Our primary objective for the current analysis was to determine accurate incidences of aLRTD, aLRTI, and pneumonia, stratified by patient age during the pandemic period. We also stratified by SARS-CoV-2 status to determine the contribution of COVID-19 to disease incidence, to establish non- SARS-CoV-2 aLRTD incidences, and whether fluctuations were observed in the context of periods of mandatory non-pharmaceutical interventions. Lastly, we sought to assess disease severity for COVID-19 LRTI, non-COVID-19 LRTI, and non-infective LRTD.

## Methods

### Ethics and permission

This is a prospective observational cohort study of adults admitted to two large university hospitals in Bristol, UK. The study was approved by the Health Research Authority Research Ethics Committee East of England, Essex, reference 20/EE/0157, ISRCTN: 17354061.

Informed consent was obtained from cognisant patients, and declarations for participation from consultees for individuals lacking capacity. If it was not practicable to approach individuals for consent, data were included using approval from the Clinical Advisory Group under section 251 of the 2006 NHS Act.

### Study design

All adults (≥18 years) admitted to both participating hospitals from 1st August 2020 to 15th November 2021, encompassing all acute secondary care in Bristol during this period, were screened for study inclusion. This time-period was selected as it encompassed the end of first wave of Wuhan, and subsequent Alpha and Delta waves in the UK, and pre-dates the emergence of the ongoing Omicron SARS-CoV-2 variant wave.^13^ Patients were screened for signs and symptoms of respiratory disease, and those with ≥ signs or a confirmed clinical or radiological aLRTD diagnosis, and disease ≤28 days in duration were included. Signs and symptoms included: documented fever (≥38°C) or hypothermia (<35.5°C); cough; increased sputum volume or discolouration; pleurisy; dyspnoea; tachypnoea; examination findings (e.g. crepitations); or, radiological changes suggestive of aLRTD in the opinion of a consultant radiologist, such as consolidation or pulmonary oedema. Patients were excluded from the study if their symptoms developed within ≥48 h of admission or within 7 days of discharge from hospital. Additionally, patients whose signs/symptoms were not attributable to aLRTD were excluded (e.g. fever and tachypnoea attributable to urosepsis). Eligible cases of aLRTD disease were then classified in to the different aLRTD subgroups (pneumonia, NP-LRTI, heart failure, chronic respiratory disease exacerbation) following the case definitions provided below and in Supplementary Table 1.

Demographic and clinical data were collected from electronic and paper patient records and recorded on an electronic clinical record form using REDCap.^14^ We collected data on co-morbidities at admission, determining Charlson co-morbidity index (CCI; with published estimates of 10-year survival)^15^ and Rockwood clinical frailty score (with a score of 5–9 indicating frailty).^16^ Vaccination records for each participant were obtained from linked general practitioner (GP) records.

### Case definitions

SARS-CoV-2 infection was defined as PCR positive test, using the established assay (Hologic Panther TMA) conducted by UKHSA diagnostic laboratories (RCP Path 2021). Patients with no molecular SARS-CoV-2 test (3.3% eligible cases) were assigned to a non-SARS-CoV-2 group. Pneumonia was classified as acute respiratory illness with confirmed radiological changes compatible with infection or when the treating clinician confirmed the diagnosis. In keeping with NICE and BTS guidelines, patients assigned a diagnosis of pneumonia were counted as a pneumonia case even if a CXR was not taken or no infiltrate was seen, due to false-negative radiology occurring, for example when consolidation is behind thoracic structures or severe dehydration. NP-LRTI was defined as the presence of signs and symptoms of acute lower respiratory tract infection in the absence of infective radiological change and a clinical diagnosis of pneumonia. Under these case definitions, any patients with aLRTD signs or symptoms due to non-infectious aLRTD would have been assigned appropriately to CRDE or heart failure groups. Full case definitions can be found in Supplementary Data 1.

### Outcomes

All-cause mortality for patients within 30-days of hospital admission was determined, in addition to hospital length of stay (days), requirement in intensive care or high-dependency unit (ICU/HDU) and length of ICU/HDU admission (days). The population of Bristol was estimated as previously described, including full methodology.^17^ Briefly, hospital admission data were linked to aggregated GP practice patient registration data within NHS Bristol, North Somerset and South Gloucestershire Clinical Commissioning Group for 2017–2019. The proportion of GP practices’ aLRTD hospitalisations that occurred at a specific study hospital was multiplied by their patient registration count for six age groups to obtain the practices’ contribution to that hospital's denominator (e.g., if 50% of GP practice admissions were at a specific study hospital among persons 50−64 years, the practice contributed half of their patients 50–64 years to the denominator). Incidence was calculated per 100,000 people from 1st August 2020 to 31st July 2021, using the case numbers (numerator) divided by population (denominator).

### Statistical analysis

The primary goal of this analysis was to report incidence rates by disease categories and SARS-CoV-2 positivity as these data are critical to inform public health decision making. All data were descriptively summarised. Comparisons between ages and CCI of SARS-CoV-2 PCR positive and SARS-CoV-2 PCR negative cases were made using Kolmogorov–Smirnov tests. Non-parametric comparisons were used as age and CCI were shown to be not normally distributed by visual inspection and Shapiro tests. Categorical data are presented as counts and percentages, and continuous data as either means with standard deviations (SD) or medians with inter-quartile (IQR) ranges. Overlapping subsets of the data pertaining to the clinical presentation of SARS-CoV-2 PCR positive and SARS-CoV-2 PCR negative cases are described using a stratified UpSet diagram.^18^ All persons aged ≥18 years contributed to the denominator for incidence estimate calculations and are reported per 100,000 persons for the aLRTD groups of the whole cohort. Analyses performed are stratified by SARS-CoV-2 PCR status, and by clinical presentation in various combinations of pneumonia, NP-LRTI, HF, and CRDE: pneumonia and NP-LRTI are mutually exclusive, but other groups could overlap. Patients that present with pneumonia or NP-LRTI are additionally grouped as having aLRTD of infectious origin (respiratory infection), whereas presentations involving only HF or CRDE are classified as having non-infectious aLRTD, comparisons of which are included in the supplementary material. Patients who were eligible for the study due to aLRTD but declined consent are analysed as undifferentiated aLRTD, also in the supplementary material. Estimates of admission incidence from weekly admission counts are made using maximum likelihood, assuming the observed case count is a Poisson distributed quantity with a time varying rate. The rate is estimated using a locally fitted order 2 polynomial using a logarithmic link function using the methods of Loader et al.^19^ All analyses were conducted using R.^20^

### Role of the funding source

The study funder had no role in data collection, but collaborated in study design, data interpretation and analysis and writing this manuscript. The corresponding author had full access to all data in the study and had final responsibility for the decision to submit for publication.

## Results

Of the 1,35,014 hospitalisations, 12,557 admissions were attributable to aLRTD, of which 12,248 (98%) consented to participate in the study. 3178 (26%) aLRTD admissions were SARS-CoV-2 infection-related, 6909 (55%) were due to infection with no evidence of SARS-CoV-2, and the remaining 2161 (17%) had no association with infection documented (Supplementary Figure 1). Overall, patients were elderly (median age 73y, IQR 25.7), with 8% residing in a care facility (Table 1). The cohort was broadly comorbid (59.8% patients had a Charlson Co-morbidity Index (CCI) ≥4) and frail (31.4%). 51.5% of patients were current or former smokers (Table 1, Supplementary Data 2).

**Table 1:** Patient characteristics of adults hospitalised with aLRTD.

		All included aLRTD		Non-infective		Confirmed SARS-CoV-2				No evidence SARS-CoV-2			
		Total		Total		Pneumonia		NP-LRTI		Pneumonia		NP-LRTI	
Characteristic	Group	N	value	N	value	N	value	N	value	N	value	N	value
Age	(mean ± SD)	12248	68·4 ± 18·6	2161	72 ± 17·3	2633	62·1 ± 17·9	545	64 ± 20·9	4028	72·9 ± 16·7	2881	66·2 ± 20
Age category	18-34	845	6·9%	111	5·1%	207	7·9%	68	12·5%	156	3·9%	303	10·5%
	35-49	1312	10·7%	134	6·2%	483	18·3%	79	14·5%	280	7·0%	336	11·7%
	50-64	2345	19·1%	358	16·6%	742	28·2%	93	17·1%	655	16·3%	497	17·3%
	65-74	2376	19·4%	457	21·1%	476	18·1%	95	17·4%	777	19·3%	571	19·8%
	75-84	2933	23·9%	564	26·1%	448	17·0%	123	22·6%	1121	27·8%	677	23·5%
	85+	2437	19·9%	537	24·8%	277	10·5%	87	16·0%	1039	25·8%	497	17·3%
Age eligible for PneumoVax	18-64	4502	36·8%	603	27·9%	1432	54·4%	240	44·0%	1091	27·1%	1136	39·4%
	65+	7746	63·2%	1558	72·1%	1201	45·6%	305	56·0%	2937	72·9%	1745	60·6%
Gender	Male	6206	50·7%	1011	46·8%	1498	56·9%	250	45·9%	2127	52·8%	1320	45·8%
	Female	6042	49·3%	1150	53·2%	1135	43·1%	295	54·1%	1901	47·2%	1561	54·2%
Ethnicity	White British	9379	76·6%	1659	76·8%	1711	65·0%	390	71·6%	3276	81·3%	2343	81·3%
	White other	341	2·8%	51	2·4%	136	5·2%	20	3·7%	74	1·8%	60	2·1%
	Mixed origin	99	0·8%	15	0·7%	27	1·0%	4	0·7%	27	0·7%	26	0·9%
	Black	237	1·9%	28	1·3%	99	3·8%	14	2·6%	54	1·3%	42	1·5%
	Asian	339	2·8%	29	1·3%	175	6·6%	23	4·2%	59	1·5%	53	1·8%
	Other race	108	0·9%	19	0·9%	49	1·9%	6	1·1%	17	0·4%	17	0·6%
	Unknown	1735	14·2%	360	16·7%	433	16·4%	88	16·1%	516	12·8%	338	11·7%
	<missing>	10	0·1%	—	—	3	0·1%	—	—	5	0·1%	2	0·1%
Care home resident	no	9970	81·4%	1881	87·0%	2172	82·5%	398	73·0%	3243	80·5%	2276	79·0%
	yes	1032	8·4%	112	5·2%	163	6·2%	45	8·3%	472	11·7%	240	8·3%
	<missing>	1246	10·2%	168	7·8%	298	11·3%	102	18·7%	313	7·8%	365	12·7%
Smoker	Unknown	1311	10·7%	219	10·1%	295	11·2%	61	11·2%	442	11·0%	294	10·2%
	Non-smoker	4470	36·5%	690	31·9%	1227	46·6%	245	45·0%	1319	32·7%	989	34·3%
	Current	1217	9·9%	196	9·1%	127	4·8%	24	4·4%	442	11·0%	428	14·9%
	Ex-smoker	5247	42·8%	1055	48·8%	984	37·4%	215	39·4%	1823	45·3%	1170	40·6%
	<missing>	3	0·0%	1	0·0%	—	—	—	—	2	0·0%	—	—
Covid vaccination	Unknown	586	4·8%	91	4·2%	159	6·0%	36	6·6%	172	4·3%	128	4·4%
	Not received	6265	51·2%	893	41·3%	1817	69·0%	278	51·0%	1928	47·9%	1349	46·8%
	Received	5396	44·1%	1177	54·5%	657	25·0%	231	42·4%	1927	47·8%	1404	48·7%
	<missing>	1	0·0%	—	—	—	—	—	—	1	0·0%	—	—
CCI	(mean ± SD)	12237	3·97 ± 2·59	2161	4·53 ± 2·5	2628	3 ± 2·5	543	3·27 ± 2·5	4026	4·52 ± 2·48	2879	3·8 ± 2·61
CURB65 score	0-Very Low	3429	28·0%	456	21·1%	1077	40·9%	200	36·7%	784	19·5%	912	31·7%
	1-Low	5534	45·2%	1096	50·7%	1043	39·6%	217	39·8%	1885	46·8%	1293	44·9%
	2-Moderate	2677	21·9%	502	23·2%	423	16·1%	108	19·8%	1081	26·8%	563	19·5%
	3-Severe	539	4·4%	97	4·5%	75	2·8%	17	3·1%	247	6·1%	103	3·6%
	4-Severe	58	0·5%	10	0·5%	10	0·4%	1	0·2%	29	0·7%	8	0·3%
	<missing>	11	0·1%	—	—	5	0·2%	2	0·4%	2	0·0%	2	0·1%
COPD	no	9121	74·5%	1574	72·8%	2314	87·9%	451	82·8%	2853	70·8%	1929	67·0%
	yes	3127	25·5%	587	27·2%	319	12·1%	94	17·2%	1175	29·2%	952	33·0%
Asthma	no	10283	84·0%	1816	84·0%	2209	83·9%	468	85·9%	3494	86·7%	2296	79·7%
	yes	1965	16·0%	345	16·0%	424	16·1%	77	14·1%	534	13·3%	585	20·3%
Bronchiectasis	no	11802	96·4%	2107	97·5%	2592	98·4%	534	98·0%	3829	95·1%	2740	95·1%
	yes	446	3·6%	54	2·5%	41	1·6%	11	2·0%	199	4·9%	141	4·9%
IHD	no	10570	86·3%	1780	82·4%	2358	89·6%	484	88·8%	3451	85·7%	2497	86·7%
	yes	1678	13·7%	381	17·6%	275	10·4%	61	11·2%	577	14·3%	384	13·3%
Hypertension	no	10484	85·6%	1829	84·6%	2324	88·3%	487	89·4%	3369	83·6%	2475	85·9%
	yes	1764	14·4%	332	15·4%	309	11·7%	58	10·6%	659	16·4%	406	14·1%
On immunosuppression	no	11159	91·1%	1948	90·1%	2489	94·5%	511	93·8%	3643	90·4%	2568	89·1%
	yes	1088	8·9%	213	9·9%	144	5·5%	34	6·2%	384	9·5%	313	10·9%
	<missing>	1	0·0%	—	—	—	—	—	—	1	0·0%	—	—
Diabetes type	None	9569	78·1%	1620	75·0%	2015	76·5%	441	80·9%	3197	79·4%	2296	79·7%
	Type 1	150	1·2%	24	1·1%	30	1·1%	10	1·8%	44	1·1%	42	1·5%
	Type 2	2528	20·6%	517	23·9%	588	22·3%	94	17·2%	786	19·5%	543	18·8%
	<missing>	1	0·0%	—	—	—	—	—	—	1	0·0%	—	—
CKD	None	9385	76·6%	1511	69·9%	2155	81·8%	447	82·0%	2993	74·3%	2279	79·1%
	Mild (CKD 1-3)	2384	19·5%	527	24·4%	405	15·4%	86	15·8%	867	21·5%	499	17·3%
	Moderate or Severe CKD (CKD 4+)	478	3·9%	123	5·7%	73	2·8%	12	2·2%	167	4·1%	103	3·6%
	<missing>	1	0·0%	—	—	—	—	—	—	1	0·0%	—	—

Patient demographics are shown for total cohort, patients with non-infective aLRTD, confirmed SARS-CoV-2 infection (total, pneumonia, NP-LRTI) and infection without SARS-CoV-2 (total, pneumonia, NP-LRTI). Full demographics are presented in Supplementary Table 1.

^a^LRTD, acute lower respiratory tract disease; CCI, Charlson comorbidity index; CKD, chronic kidney disease; COPD, chronic obstructive pulmonary disease; IHD, ischaemic heart disease; NP-LRTI, non-pneumonic lower respiratory tract infection; PCR, polymerase chain reaction; SD, standard deviation.

^†^In the UK, patients aged ≥65 years are eligible for Pneumococcal vaccination (PneumoVax®, PPV23) once, and annual influenza vaccine.

*Hypertension was only included if causing other cardiac complications.

**Chronic kidney disease (CKD) was classified as mild if stage 1-3; moderate/severe if stage 4-5, end-stage renal failure or there was dialysis dependence.

Additional data are located in Supplementary Table 2.

**Figure 1:**
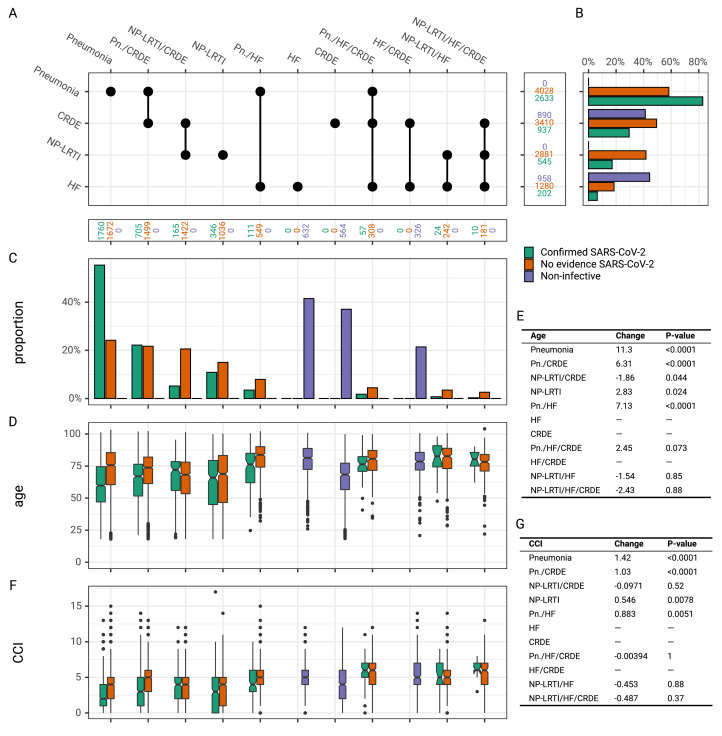
**Summary of aLRTD incidence between 1st August 2020 and 15th November 2021**. Panel A shows categories with combinations of having a single aLRTD phenotype (e.g., pneumonia alone), two phenotypes (e.g., pneumonia and CRDE), or three phenotypes (e.g., pneumonia, CRDE and HF); the numbers along the axes show counts for each phenotype, both singly and in combination, stratified by SARS-CoV-2 PCR status. 640/6661 (9.6%) cases of pneumonia were not radiologically confirmed [423/6661 (6.4%) with no consolidation/infiltrate and 217/6661 (3.3%) with no radiology performed]. Panel B shows the proportion of cases with each single aLRTD phenotype out of the total number of cases in each strata. Where cases have multiple phenotypes they are counted once for each phenotype, hence proportions do not add up to 100%. Panel C shows the proportion of cases with every combination of aLRTD phenotypes, stratified by SARS-CoV-2 status. In this panel each case is counted only once and hence proportions do add up to 100%. Panels E and F show boxplot summaries of the distributions for key patient indicators in each phenotype combination category: E) age, and F) CCI score, stratified by SARS-CoV-2 PCR status. Panels D and G indicate differences in these key indicators between SARS-CoV-2 PCR positive and negative patients for D) age, and G) CCI score, in tabular form. *P*-values are the result of 2 sided Kolmogorov–Smirnov significance tests. CCI, Charlson Comorbidity Index; CRDE, chronic respiratory disease exacerbation; HF, heart failure; NP-LRTI, non-pneumonic lower respiratory tract infection; Pn, pneumonia.

The proportion of aLRTD hospitalisations due either to SARS-CoV-2 or other infections was high (10,087/12,557, 81%). SARS-CoV-2 infection usually presented as pneumonia alone (Figure 1C) and pneumonia was more frequent in patients with SARS-CoV-2 infection than those infection cases who had negative SARS-CoV-2 PCR results (Figure 1B). Similarly, SARS-CoV-2 infection more commonly presented as pneumonia than as NP-LRTI in patients who had associated HF, CRDE or both as a component of their aLRTD. The SARS-CoV-2 patients presenting with pneumonia alone were, on average, 11.3 years younger than SARS-CoV-2 PCR negative pneumonia patients (*P*<0.001) (Figure 1E, D), and had fewer comorbidities as indicated by an average CCI score lower by 1.42 (*P* <0.001) (Figure 1F, G). Similar significant, although smaller, differences were observed in patients with pneumonia combined with other factors such as HF and CRDE.

COVID-19 morbidity and mortality were considerable: median hospital length of stay was 5 days (IQR 9.0) and increased with patient age; 427/12,557 (3%) patients required ICU/HDU care with median ICU/HDU admission duration of 7 days (IQR 11.0), and 1146/12,557 (9%) patients died within 30 days of admission (Table 2, Supplementary Table 3). Mortality increased with patient age: hospitalised patients aged 18–34 years had a 30-day mortality rate <1.0% compared to 17.3% in those ≥85 years. Among all aLRTD subgroups, elderly patients with COVID-19 had a higher 30-day mortality than those with non-SARS-CoV-2 aLRTD (Table 2), e.g. 23.5% [20.2–27.1%] mortality for confirmed SARS-CoV-2 versus 11.8% [10.4–13.4%] for non-SARS-CoV-2 infective aLRTD in 74–85 age category (Supplementary Table 5; *P*<0.001) . The interquartile ranges for length of hospital admission for non-SARS-CoV-2 infective aLRTD overlapped with those of SARS-CoV-2 infection across all patient age groups (Table 2), however a statistically significant difference in length of stay was observed over all age groups, with confirmed SARS-CoV-2 patients being in hospital longer than non-SARS-CoV-2 infective aLTRD patients. For example, the length of stay in 74–85-year-old patients increased from 5.5 [IQR 2 – 12] with non-SARS-CoV-2 aLRTD to 8 days [IQR 4 – 15] with confirmed SARS-CoV-2 (Supplementary Table 4; *P*<0.01).

**Table 2:** Outcomes of patients with non-infective aLRTD, proven SARS-CoV-2 aLRTD, and other infective aLRTD.

	All included aLRTD	Non-infective	Confirmed SARS-CoV-2		No evidence SARS-CoV-2	
	Total	Total	Pneumonia	NP-LRTI	Pneumonia	NP-LRTI
Length of stay	Median [IQR]	Median [IQR]	Median [IQR]	Median [IQR]	Median [IQR]	Median [IQR]
Overall	5 [2 – 11]	4 [1 – 10]	7 [3 – 13]	4 [1 – 9]	6 [2 – 12]	3 [1 – 7]
18-34	2 [0 – 4]	1 [0 – 3]	3 [1 – 6]	2 [1 – 4]	2 [1 – 5·2]	1 [0 – 3]
35-49	3 [1 – 7]	1 [0 – 4]	5 [3 – 9]	1 [0·5 – 5]	3 [1 – 7·2]	1 [0 – 4]
50-64	5 [2 – 9]	3 [1 – 8]	7 [4 – 12]	3 [1 – 7]	5 [2 – 10]	2 [1 – 5]
65-74	5 [2 – 11]	4 [2 – 9]	9 [4 – 16]	4 [2 – 8·8]	6 [2 – 12]	3 [1 – 8]
75-84	6 [2 – 13]	5 [2 – 12]	9 [4 – 15]	6 [3 – 14]	6 [3 – 14]	4 [1 – 9]
85+	7 [3 – 15]	6 [2 – 13]	10 [4 – 20]	10 [4 – 20]	7 [3 – 15]	5 [2 – 12]
18-64	3 [1 – 8]	2 [1 – 6]	6 [3 – 10]	2 [1 – 5]	4 [1 – 9]	2 [0 – 4]
65+	6 [2 – 13]	5 [2 – 11]	9 [4 – 17]	6 [2 – 14]	6 [3 – 14]	4 [2 – 9]
ICU admission	N (%)	N (%)	N (%)	N (%)	N (%)	N (%)
Overall	427/12248 (3·5%)	27/2161 (1·2%)	251/2633 (9·5%)	7/545 (1·3%)	119/4028 (3·0%)	23/2881 (0·8%)
18-34	29/845 (3·4%)	1/111 (0·9%)	11/207 (5·3%)	1/68 (1·5%)	13/156 (8·3%)	3/303 (1·0%)
35-49	74/1312 (5·6%)	0/134 (0·0%)	57/483 (11·8%)	0/79 (0·0%)	14/280 (5·0%)	3/336 (0·9%)
50-64	151/2345 (6·4%)	7/358 (2·0%)	101/742 (13·6%)	3/93 (3·2%)	31/655 (4·7%)	9/497 (1·8%)
65-74	110/2376 (4·6%)	13/457 (2·8%)	60/476 (12·6%)	1/95 (1·1%)	34/777 (4·4%)	2/571 (0·4%)
75-84	57/2933 (1·9%)	5/564 (0·9%)	22/448 (4·9%)	2/123 (1·6%)	22/1121 (2·0%)	6/677 (0·9%)
85+	6/2437 (0·2%)	1/537 (0·2%)	0/277 (0·0%)	0/87 (0·0%)	5/1039 (0·5%)	0/497 (0·0%)
18-64	254/4502 (5·6%)	8/603 (1·3%)	169/1432 (11·8%)	4/240 (1·7%)	58/1091 (5·3%)	15/1136 (1·3%)
65+	173/7746 (2·2%)	19/1558 (1·2%)	82/1201 (6·8%)	3/305 (1·0%)	61/2937 (2·1%)	8/1745 (0·5%)
ICU length of stay	Median [IQR]	Median [IQR]	Median [IQR]	Median [IQR]	Median [IQR]	Median [IQR]
Overall	7 [4 – 15]	5 [3 – 9]	9 [5 – 16]	6 [4·5 – 11]	6 [3 – 12]	3 [1·5 – 5]
18-34	3 [2 – 7]	6 [6 – 6]	4 [2·5 – 11]	1 [1 – 1]	3 [2 – 11]	3 [2 – 3·5]
35-49	7·5 [4 – 15]	―	7 [4 – 14]	―	9·5 [4·2 – 15]	3 [2·5 – 3]
50-64	8 [4 – 16]	3 [2 – 4]	9 [5 – 18]	15 [10 – 22]	7 [3·5 – 16]	3 [1 – 5]
65-74	8 [4 – 17]	7 [4 – 28]	10 [6 – 17]	4 [4 – 4]	6 [3 – 13]	7·5 [7·2 – 7·8]
75-84	7 [5 – 12]	5 [5 – 8]	11 [7·2 – 15]	6 [5·5 – 6·5]	6 [4 – 10]	3 [2 – 4·8]
85+	3 [2 – 4]	4 [4 – 4]	―	―	2 [2 – 4]	―
18-64	7 [4 – 15]	3·5 [2 – 4·5]	8 [4 – 16]	10 [4·8 – 18]	7 [3 – 15]	3 [1 – 4·5]
65+	8 [4 – 14]	6 [4 – 14]	10 [6 – 16]	5 [4·5 – 6]	6 [3 – 11]	4·5 [2 – 7·2]
All-cause mortality	N (%)	N (%)	N (%)	N (%)	N (%)	N (%)
Overall	1146/12248 (9·4%)	124/2161 (5·7%)	339/2633 (12·9%)	32/545 (5·9%)	543/4028 (13·5%)	108/2881 (3·7%)
18-34	3/845 (0·4%)	0/111 (0·0%)	0/207 (0·0%)	0/68 (0·0%)	2/156 (1·3%)	1/303 (0·3%)
35-49	20/1312 (1·5%)	1/134 (0·7%)	11/483 (2·3%)	0/79 (0·0%)	8/280 (2·9%)	0/336 (0·0%)
50-64	93/2345 (4·0%)	11/358 (3·1%)	35/742 (4·7%)	2/93 (2·2%)	38/655 (5·8%)	7/497 (1·4%)
65-74	209/2376 (8·8%)	19/457 (4·2%)	79/476 (16·6%)	4/95 (4·2%)	82/777 (10·6%)	25/571 (4·4%)
75-84	390/2933 (13·3%)	44/564 (7·8%)	121/448 (27·0%)	13/123 (10·6%)	172/1121 (15·3%)	40/677 (5·9%)
85+	431/2437 (17·7%)	49/537 (9·1%)	93/277 (33·6%)	13/87 (14·9%)	241/1039 (23·2%)	35/497 (7·0%)
18-64	116/4502 (2·6%)	12/603 (2·0%)	46/1432 (3·2%)	2/240 (0·8%)	48/1091 (4·4%)	8/1136 (0·7%)
65+	1030/7746 (13·3%)	112/1558 (7·2%)	293/1201 (24·4%)	30/305 (9·8%)	495/2937 (16·9%)	100/1745 (5·7%)

*Number of ICU/HDU cases is numerator; patients (n) in corresponding age group is denominator.

**Cases with survival days ≤30 days following hospitalization is numerator; patients (n) in corresponding age group is denominator.

†In the UK, patients aged ≥65 years are eligible for Pneumococcal vaccination (PneumoVax®, PPV23) once and annual influenza vaccine.

aLRTD, acute lower respiratory tract disease; ICU, intensive care unit; IQR, interquartile range; NP-LRTI, non-pneumonic lower respiratory tract infection.

Additional data are located in Supplementary Table 3.

**Figure 2:**
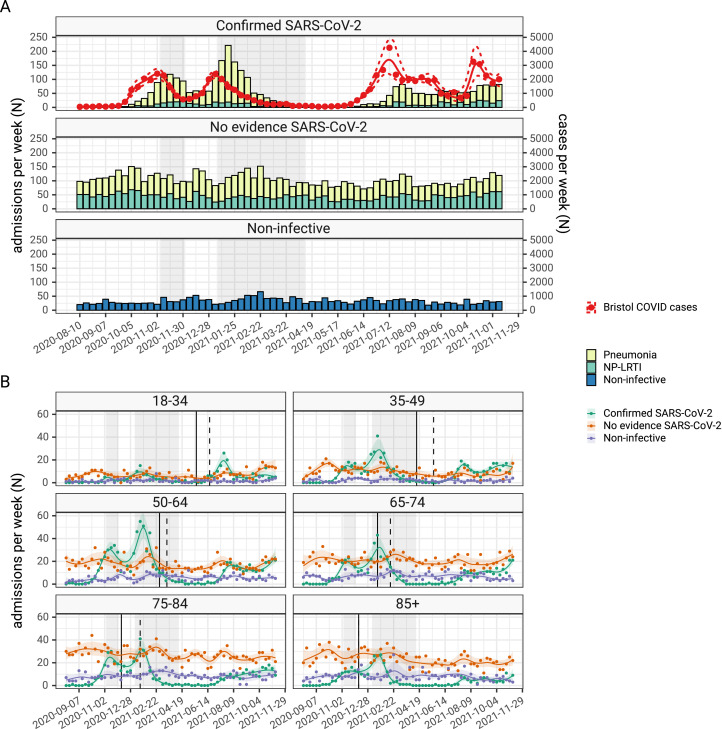
**Hospitalisations with aLRTD, by primary clinical presentation (non-infective, SARS-CoV-2 infection and infection with no evidence of SARS-CoV-2) and age group**. (A) The upper panel shows the weekly number of hospital admissions associated with positive SARS-CoV-2 PCR results taken at the time of admission, as a bar chart. For comparison the red line shows the weekly number of SARS-CoV-2 cases in the immediate locality of the study sites. In the second panel we show the remaining non-SARS-CoV-2 infection aLRTD admissions, stratified by primary clinical presentation, and in the 3^rd^ panel non-infective aLRTD admissions (including primary presentations with heart failure and/or exacerbation of chronic respiratory disease). Pneumonia cases are shown in yellow, and NP-LRTI in green. (B) Points represent weekly counts of aLRTD admissions. Estimates of the underlying incidence rates are shown as continuous lines, assuming the admissions follow a Poisson distribution with a time varying rate, using a locally fitted polynomial in time, using a maximum likelihood method. The earliest date that the COVID-19 vaccination program opened to any people in each age group is marked on each panel with a solid vertical line, and the date by which all people in the age group were eligible for vaccination by a dashed vertical line (where different) (Supplementary Table 5). Grey bars in the background indicate periods when non-pharmaceutical interventions were in place to control the spread of SARS-CoV-2. (For interpretation of the references to color in this figure legend, the reader is referred to the web version of this article.)

There were considerable fluctuations in hospital admissions due to COVID-19 during the course of the study, which followed community COVID-19 disease incidence and aligned with different waves of lockdown in the UK (Figure 2A). The age distributions of patients admitted during the two waves of COVID-19 reported here were different. Admissions earlier in the study (Alpha wave) were predominantly older adults whereas later time periods (Delta wave) saw increasing numbers of younger adults hospitalised. Hospitalisations due to COVID-19 increased across all age groups during the COVID-19 waves until lockdown measures were introduced, following which they decreased (Figure 2B). Following the vaccine programme roll out in late 2020, COVID-19 hospitalisations in prioritised older age groups began to fall. The number of COVID-19 hospitalisations among younger adults fluctuated during the study period, with COVID-19 overall being the predominant cause of hospital treatment for respiratory disease among these patients (aged 18–35) (Figure 2B, 5).

In contrast, rates of admission with non-SARS-CoV-2 respiratory infection (pneumonia or NP-LRTI) showed much less variation during the study period (Figure 2A) and did not follow community COVID-19 incidence. Hospitalisation rates increased with patient age (Figure 2B), with disease predominantly affecting those over 65 years old. No clear associations between periods of implementation of lockdown measures and hospitalisation rates due to non-SARS-CoV-2 pneumonia and NP-LRTI were observed.

**Figure 3:**
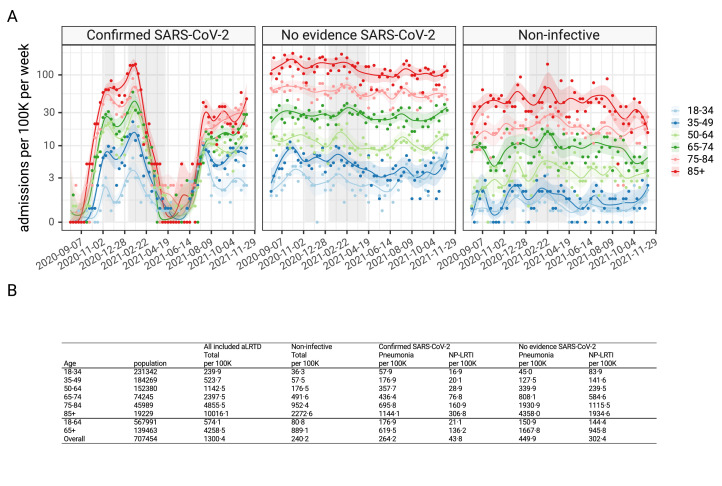
**Incidence per 100,000 population per week by age group for non-infective aLRTD, PCR positive SARS-CoV-2 aLRTD, and infection with no evidence of SARS-CoV-2**. (A) As in Figure 3 estimates of the underlying incidence rates are shown as continuous lines. Grey bars in the background indicate periods when non-pharmaceutical interventions were in place to control the spread of SARS-CoV-2. (B) aLRTD cumulative hospital cases (per 100,000 people) in adults in Bristol, UK over 12 months (August 2020–July 2021). Additional data are located in Supplementary Table 4. aLRTD, acute lower respiratory tract disease; NP-LRTI, non-pneumonic lower respiratory tract infection; PCR, polymerase chain reaction.

While COVID-19 contributed substantially to respiratory infection incidence during this pandemic period, non-COVID-19 cases contributed more to both pneumonia and NP-LRTI disease: annual incidences of the latter among adults aged ≥18 years were 450 and 302 per 100,000 population, respectively (Figure 3, Supplementary Table 4). Non-SARS-CoV-2 disease was the most common cause of NP-LRTI in all age groups. Although non-SARS-CoV-2 pneumonia incidence was lower than COVID-19 among adults aged <65 years, among those aged 65–74, 75–84, and 85+ years it was 1.9, 2.8, and 3.8-fold higher, respectively. Weekly incidence per 100,000 population was higher for SARS-CoV-2 PCR negative aLRTD in hospitalised patients than for SARS-CoV-2 PCR positive disease across all age groups (Figure 3). Incidence rose with patient age and the incidence of SARS-CoV-2 infection in hospitalised patients increased as new variants emerged and fell, following non-pharmaceutical interventions.

## Discussion

This two-site single-centre study conducted within a defined geographical area describes aLRTD during part of the COVID-19 pandemic, covering the end of the first wave of the Wuhan strain, through subsequent waves of the Alpha and Delta variants and pre-dates the emergence of the Omicron variant. By conducting comprehensive surveillance of all aLRTD, we determined the incidence and severity of both COVID-19 and non- SARS-CoV-2 disease in individuals needing hospital care in this population.

Notwithstanding the emergence of COVID-19, 56% (6909/12,248) of aLRTD was due to non-SARS-CoV-2 infection, despite public health interventions to reduce hospitalisations and NHS-burden whilst vaccination was rolled out, and the burden of hospitalised non-SARS-CoV-2 infection was greater than that of SARS-CoV-2 infection. Moreover, annual aLRTD, NP-LRTI, and pneumonia incidences were comparable to a pre-pandemic retrospective analysis undertaken at one of the study hospitals (pneumonia and NP-LRTI 591 and 739/100,000 versus 714 and 346/100,000 people found in this study, Supplementary Table 4).^21^ Not only did non- SARS-CoV-2 pneumonia and NP-LRTI not substantially decline despite public health interventions to reduce hospitalisations and NHS burden, but compared to previous pre-pandemic UK studies^3^, ^4^, ^5^^,^^21^^,^^22^ we report higher pneumonia incidences. For example, compared to a recent study from Nottingham reporting data from 2013 to 2018, non-COVID-19 pneumonia incidences from our study were 2.6 to 4.9-fold, 3.4 to 5.0-fold, and 2.9 to 8.6-fold higher for, respectively, persons age 65–74y, 75–84y, and ≥85y. The precise reasons for these discordant results is beyond the scope of our current evaluation but may relate to the methodology we employed to conduct comprehensive, prospective, population-based surveillance to identify every adult hospitalised with aLRTD. Further, the difference in estimated incidence may be explained in part by the inclusion criteria of this study, which allowed for patients with atypical symptoms and those with a clinical diagnosis of pneumonia.

Non-SARS-CoV-2 respiratory infection did not show much seasonal variation and did not follow trends in COVID-19-related admissions. Variation in the treatment preference, admission threshold,^23^^,^^24^ or other confounders may have affected non-SARS-CoV-2 disease admissions more than COVID-19 admissions, and the incidence estimate calculated here may therefore be lower for non-SARS-CoV-2 aLRTD than if non-pharmaceutical interventions had not been implemented, as supported by previous studies showing that respiratory pathogen infections dramatically decreased during the pandemic.^9^^,^^25^^,^^26^ Consequently, incidences for non-SARS-CoV-2 aLRTD disease may increase in the future as non-pharmaceutical interventions for reducing SARS-CoV-2 transmission are relaxed. Ongoing accurate and systematic surveillance will be needed to determine how disease incidences and risk groups change as the current pandemic evolves, and these data will be available in coming periods during this ongoing study. Even if incidence estimates calculated in this study change during future years, they provide valuable insight into the burden of acute respiratory infection and how effective public health measures, including increased use of vaccination in adults, might be used to reduce disease.

Overall, 26% (3178/12,248) of adults hospitalised with aLRTD had SARS-CoV-2 infection during this period of the pandemic, highlighting the significant impact of COVID-19 on total respiratory infection burden and healthcare resource usage. There was considerable variability in rates of hospital admission due to COVID-19 throughout the study and successive national lockdowns appear to have been effective in reducing these hospital admissions. Following the COVID-19 vaccination programme implementation, COVID-19 admissions declined, with no further surges observed as successive patient age groups were included (Figure 2). Although SARS-CoV-2 infection disproportionately affected older adults, a substantial number of younger patients required both hospitalisation and ICU treatment due to COVID-19, reinforcing the observation that this disease is not always mild in young individuals.^27^

Aligning with previous literature, aLRTD disease of all causes disproportionately affected older people, highlighting an incidence in people over 85 years old (10,016.1 cases per 100,000 per year) which was 42-times higher than that seen in people aged 18–34 (239.9 cases per 100,000 per year) (Figure 3). 20% of patients aged ≥65 years admitted with pneumonia died within 30 days (Supplementary Table 3), higher than that observed nationally for hip fracture (5.36%)^28^ and myocardial infarction (15.6%),^29^ and comparable to stroke (21.0% female, 19.8% males).^30^ The non-SARS-CoV-2 pneumonia mortality of 13.5% in this study (Table 2) was comparable to that reported in a pre-pandemic national audit, which showed overall 30-day pneumonia mortality of 14.6%, but did not specify mortality by age group and excluded NP-LRTI.^31^ We found the 30-day mortality from non-SARS-CoV-2 NP-LRTI was lower in all patient age groups than that for patients with pneumonia, HF and CRDE. We also documented among persons age ≥65 years that disease severity was worse with SARS-CoV-2 versus non-SARS-CoV-2 respiratory infection. Whether the acute severity of SARS-CoV-2 infection translates into greater long-term morbidity, such as increased risk of subsequent aLRTD events, will be a subject for future evaluations from AvonCAP. Interestingly, 29.5% of patients admitted with pneumonia had a CURB-65 score of 0, also in line with findings from a pre-pandemic national audit, which was unable to elucidate reasons for the decrease in severity scores on admission.^31^ Future evaluation from AvonCAP may provide some reason for the increasing rate of hospitalisation with low severity score respiratory infection.

This study has many strengths. Firstly, it was undertaken prospectively and by screening hospital admissions for signs/symptoms of aLRTD. Prospective, comprehensive case ascertainment within a defined geographical area remains the gold standard epidemiological methodology for estimating disease incidence. This study did not rely on ICD-10 coding or solely on national data-linkage and were able to assess each case individually and gather complete data. Secondly, we were able to include adults hospitalised with aLRTD through a consultee if patients lacked capacity and by using specific authorisation to collected data without consent, thus ensuring full ascertainment of cases. This ensured that patients lacking capacity, such as those severely ill or with advanced dementia or other frailty, were not under-represented in this study. This study was conducted at two hospitals which provide all acute secondary medical care for the same city and time-period to undertake comprehensive surveillance in a defined geographical area with a well-defined local population, and therefore provide an accurate estimate of disease incidence and severity. The study hospitals are within a few miles of each other, with overlapping patient catchments and clinicians rotate between the two healthcare facilities. Whilst there are some differences in the demographics of patients admitted to each hospital (Supplementary Data 5 and 6), we anticipate that any independent effect of hospital site on patient outcome (beyond differences associated with patient demographics) is likely to be small. The medical records were linked with community records to obtain detailed and accurate data for each study participant. Finally, we calculated incidence using a denominator derived from GP records and hospital utilization data, providing increased accuracy compared to population estimates based on assumptions using local geographic boundaries and their corresponding census data.^17^

There are also some limitations to this study, which was conducted over 15-months overall and 12-months for incidence determination, and is therefore only able to report incidence within this time period. The incidence calculations and disease severity determinations were measured during the COVID-19 pandemic and were undoubtedly affected by the emergence of this new respiratory pathogen, public health interventions such as social distancing and vaccination and other factors that may be difficult to quantify. It is difficult to determine whether access to healthcare changed during this period; for example, clinicians or patients may have preferred treatment at home, which may have affected severity assessment and admission rates, and therefore our observed incidence. However, the study hospitals reduced elective admissions and undertook measures to avoid exceeding maximum capacity; therefore, capacity is unlikely to have limited acute admissions and thus affected our incidence calculation. Whilst we assessed aLRTD at both acute care NHS hospitals in Bristol, we cannot be sure that it is generalisable to other cities and regions. Furthermore, this cohort is predominantly (75*.*9%) White-British and therefore aLRTD disease in cohorts with different ethnicity may vary from that reported here. 309 (2%) patients actively declined to participate in the study, so we can be certain they had aLRTD but could not ascertain any additional data. Previous exposure to SARS-CoV-2 could not be determined for study participants, and this may have impacted on our findings, although the magnitude of any such effect is unclear. Additionally, to prioritise SARS-CoV-2 testing, study hospitals undertook limited microbiological testing for other respiratory pathogens and, using standard-of-care results, we are unable to comment on the microbial aetiology of non-SARS-CoV-2 respiratory infection. Whilst we describe differences in patients admitted with COVID-19, non-SARS-CoV-2 infection and non-infective aLRTD, further analyses, including adjusting for potential confounders, is needed to fully explore the reasons for these differences.

These results, in the context of an ageing population with increasing comorbid disease, coupled with the emergence of COVID-19 and potentially future novel respiratory pathogens, demonstrate the significant burden of acute respiratory disease and infection, and demonstrate the importance of consideration of the impact of aLRTD on healthcare systems. Our findings demonstrate the significant contribution of non-SARS-CoV-2 respiratory infection to total aLRTD burden in hospitalisations, and highlight the importance of not overlooking the multiple causes of respiratory infection during the COVID-19 pandemic. Providing appropriate care for adults with aLRTD and its disease subsets will require appropriate healthcare planning and resource allocation. Appropriate public health measures to reduce respiratory disease burden as well as improve patient outcomes should be implemented. In the short-term social distancing and face masks, which are effective in reducing pathogen transmission and aLRTD incidence^8^, should be considered. Vaccination to prevent adult respiratory disease is likely to be one of the most effective available short-term and long-term strategy to reduce this substantial public health burden, alongside reductions in risk factors such as cigarette smoking and ambient air pollution.

## Contributors

CH, RC, EB, JS, JO, AV, BG, LD and AF generated the research questions and analysis plans. The AvonCAP research team, CH, JK, AM, MGG, ZSB, and JK were involved in data collection. CH, MGG, ZSB and AF verified the data. CH, RC, EB, JS JC, AV, SV, SG, RH, JMM, GE, NM, LD and AF undertook data analysis. All authors contributed to preparation of the manuscript and its revisions before publication. BG and AF provided oversight of the research.

## Data sharing statement

The data used in this study are sensitive and cannot be made publicly available without breaching patient confidentiality rules. Therefore, individual participant data and a data dictionary are not available to other researchers.

## Declaration of interests

CH is Principal Investigator of the Avon CAP study which is an investigator-led University of Bristol study funded by Pfizer and has previously received support from the NIHR in an Academic Clinical Fellowship. JO is a Co-Investigator on the Avon CAP Study. LD is further supported by UKRI through the JUNIPER consortium (grant number MR/V038613/1), MRC (grant number MC/PC/19067), EPSRC (EP/V051555/1 and The Alan Turing Institute, grant EP/N510129/1). AF is a member of the Joint Committee on Vaccination and Immunization (JCVI) and chair of the World Health Organization European Technical Advisory Group of Experts on Immunization (ETAGE) committee. In addition to receiving funding from Pfizer as Chief Investigator of this study, he leads another project investigating transmission of respiratory bacteria in families jointly funded by Pfizer and the Gates Foundation and is an investigator in trials of COVID19 vaccines including ChAdOx1nCOV-19, Janssen and Valneva vaccines. EB, JS, JC, SG, RH, SV, AV, JM, GE, and BG are employees of Pfizer and own Pfizer stock. The other authors have no relevant conflicts of interest to declare. The AvonCAP study is a University of Bristol sponsored study which is investigator-led, and funded under a collaborative agreement by Pfizer Inc.

## Appendix Supplementary materials

Image, application 1

Image, application 2
