# Supplementary figures and images for: Incidence of community acquired lower respiratory tract disease in Bristol, UK during the COVID-19 pandemic: A prospective cohort study
Source: Lancet Reg Health Eur. 2022 Aug 8;21:100473. doi: 10.1016/j.lanepe.2022.100473 (PMC9359590; doi:10.1016/j.lanepe.2022.100473)

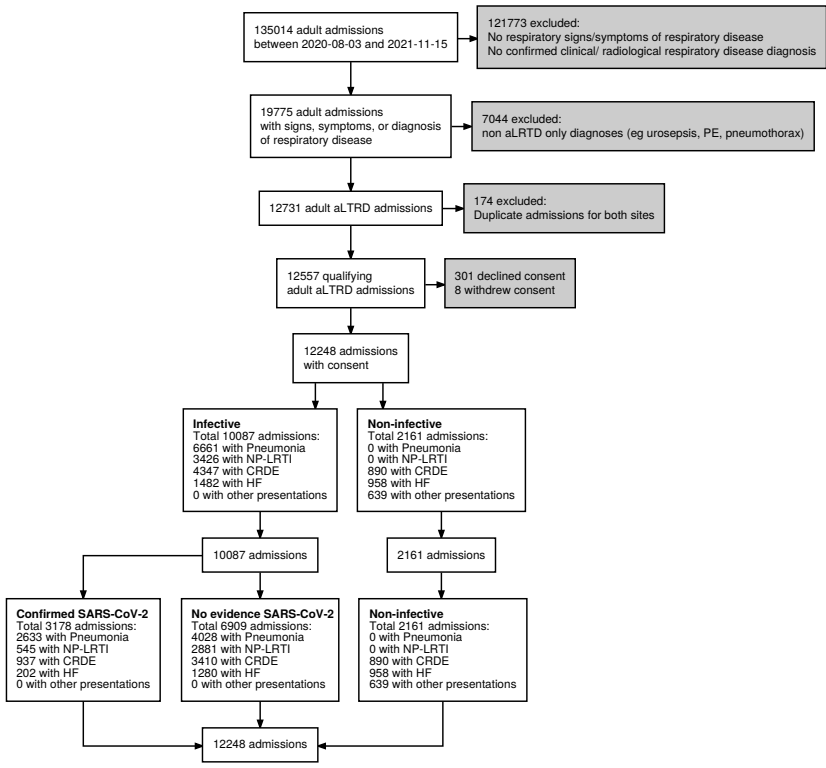

Supplement: Supplementary file 1 [file mmc1.pdf]
